# Effects of personality on the developmental trajectories of academic burnout among Korean medical students

**DOI:** 10.7717/peerj.10362

**Published:** 2020-11-12

**Authors:** Han Chae, C. Robert Cloninger, Soo Jin Lee

**Affiliations:** 1 School of Korean Medicine, Pusan National University, Busan, Korea; 2 Department of Psychiatry, School of Medicine, Washington University in St. Louis, St. Louis, MO, USA; 3 Department of Psychology, Kyungsung University, Busan, South Korea

**Keywords:** Academic burnout, Developmental trajectory, General Growth Mixture Model (GGMM), Maslach Burnout Inventory-Student Survey (MBI-SS), Medical student, Temperament and Character Inventory (TCI)

## Abstract

**Background:**

Medical students have a high risk of burnout from tremendous academic stress, and previous cross-sectional studies have explained this risk from the personality perspective. However, the relationship between complex personality profiles and developmental trajectory of burnout has not been delineated yet.

**Methods:**

The longitudinal changes in burnout were measured by the Maslach Burnout Inventory-Student Survey (MBI-SS) at baseline (1st week), mid-term (9th week), and end-term (17th week), and personality was examined at baseline using the Temperament and Character Inventory (TCI). Latent trajectory groups based on the MBI-SS total scores were extracted using the General Growth Mixture Model (GGMM), and significant differences in personality profiles among the latent groups were identified using profile analysis and Analysis of Variance.

**Results:**

Three burnout trajectory groups of high-increasing (HI), moderate-increasing (MI), and low-stable (LS) were identified, and these groups had significantly different TCI subscale profiles. The HI group had the highest score in Harm-Avoidance (HA) and lowest score in Self-Directedness (SD), and the MI group had a higher score in HA and lower scores in SD and Cooperativeness (CO) when compared to the LS group with the lowest score in HA and highest scores in SD and CO.

**Conclusion:**

The current study showed that the HA, SD, and CO subscales of the TCI might explain the longitudinal development of academic burnout in medical students. Prevention of burnout and promotion of well-being in medical education concerning personality are discussed.

## Introduction

The majority of medical students experience academic burnout from a highly competitive environment, tremendous volumes of learning, frequent examinations, concerns about academic achievement and career uncertainty, lack of time for rejuvenation, intimidating and unfavorable environment, and fear of academic failure and grade retention (Cecil et al., 2014; Chang, Kim & Kim, 2012; Erschens et al., 2019; Frajerman et al., 2019; Guthrie et al., 1998; Han et al., 2009; Ishak et al., 2013; Kim et al., 2015; Lee et al., 2012; Lee, Choi & Chae, 2017). They are reported to suffer from many problems including anxiety, depressive symptoms, alcohol abuse, poor immune function, and low quality of life during the school years (Frajerman et al., 2019; Kim, Shin & Kang, 1995; Shapiro, Shapiro & Schwartz, 2000) along with poor doctor-patient relationship, low quality of clinical practice, frequent sick leaves, and mental health problems during clinical practice (Guthrie et al., 1998; Yoong et al., 1999).

Cross-sectional studies have revealed associations between academic burnout and personality traits, such as neuroticism, extraversion, and openness to experience of the NEO-Personality Inventory (NEO-PI) (Huebner & Mills, 1994; Zellars, Perrewe & Hochwarter, 2000). However, studies focusing on individual differences in vulnerability and resilience to burnout from the personality perspective are scarce (Eley et al., 2016; Lee, Choi & Chae, 2017), and research examining the causality of academic burnout in medical professionals has been insufficient (Chae & Lee, 2017; Kim et al., 2015; Lee, Choi & Chae, 2017).

That is, the burnout studies have mainly focused on a single factor using cross-sectional measures (Mäkikangas & Kinnunen, 2016) only providing a simplified sketch of burnout symptoms and failed to reveal intrinsic and long-lasting causes trapping a person in the burnout situation and facilitating its development (Hultell, 2011; Lee, Choi & Chae, 2017). Therefore, longitudinal studies on person-centered psychological profiles are required to understand complex and holistic characteristics of individuals (Cloninger & Zwir, 2018; Stoyanov & Cloninger, 2012) and its continuing influence on the pathophysiology of burnout.

The Temperament and Character Inventory (TCI) provides two interrelated temperament and character domains which can explain the vulnerability and resilience to psychological issues based on a person-centered biopsychological theory of Cloninger (Cloninger & Svrakic, 1997; Cloninger, Svrakic & Przybeck, 1993). Temperament is a biological aspect of personality that remains stable throughout life, and character is a value of person to self and society that is shaped and developed through interactions with the environment (Cloninger et al., 2019a). Furthermore, previous studies (Eley et al., 2016; Lee, Choi & Chae, 2017; Stoyanov & Cloninger, 2012) have repeatedly shown that the personality profile combining Harm-Avoidance (HA) temperament and Self-Directedness (SD) character determines the susceptibility to psychopathology and resilience of biopsychological well-being.

Previous studies on psychological profiles of medical students (Eley et al., 2016; Lee, Choi & Chae, 2017) have considered personality factors of TCI as predictors of burnout by using regression and profile analysis, however limited studies have examined its developmental influences and psychological characteristics of individuals together across time. Recently, the person-centered approach of the General Growth Mixture Model (GGMM) was reported to be useful for analyzing longitudinal data to present heterogeneity in developmental trajectories (Hultell, Melin & Gustavsson, 2013; Martinent, Louvet & Decret, 2016; Salmela-Aro & Upadyaya, 2014). The GGMM captures the mean growth curve for each latent class and individual variations around it at multiple time points, and may provide clinical evidence for theories of multiple developmental pathways (Mäkikangas & Kinnunen, 2016; Muthen & Muthen, 2000).

Comprehensive understanding of the effects of the personality complex on developmental changes in burnout symptoms might be attained by using the personality theory of innate temperament and mature character behind the TCI and the person-centered approach of the GGMM that considers an individual as a functioning whole.

Therefore, the current study aimed to extract the developmental latent groups based on the Maslach Burnout Inventory-Student Survey (MBI-SS) total score throughout a semester of medical school using the GGMM and to identify the psychological characteristics of each latent group measured by the TCI at the beginning of the semester using profile analysis and ANOVA (Cloninger & Zwir, 2018). It would show the relationship between vulnerable academic burnout trajectory and the distinct combination of HA and SD, verifying the results of the cross-sectional findings. Also, it might provide a foundation for establishing medical education and clinical training program considering the psychological well-being of medical professionals (Cloninger & Zohar, 2011; Eley et al., 2016).

## Methods

### Participants and procedures

One hundred eighty-four students from years 1 to 4 of the School of Korean Medicine were asked to complete the MBI-SS and TCI assessing their academic burnout and personality characteristics, respectively, in 2014. The TCI and MBI-SS were measured during the 1st week of the semester as baseline. Thereafter, the MBI-SS was measured again at the 9th week of the semester as mid-term and 17th week of the semester as end-term. The Institutional Review Board of the School of Korean Medicine (KMEDIRG2013-01) approved the current study, and all participants provided the informed written consent in advance.

### Measures

#### Maslach Burnout Inventory-Student Survey

Academic burnout of medical students was measured using the MBI-SS comprising three subscales of Emotional Exhaustion, Cynicism, and Inefficacy, first implemented for university students in 2002 (Schaufeli et al., 2002).

The Emotional Exhaustion subscale represents feeling of exhaustion due to study demands. The Cynicism subscale implies distanced and detached attitude towards the study and school life itself. The Inefficacy subscale represents a feeling of academic incompetency and continued ineffectiveness at school, and is a reverse coding of Efficacy score in the original MBI developed by Maslach and Jackson (Lee, Choi & Chae, 2017; Maslach & Jackson, 1981). The MBI-SS total score is a sum of the Emotional Exhaustion, Cynicism, and Inefficacy subscales, and it’s high score represents the high degree of burnout such as emotionally exhausted from the prolonged burden of learning, feeling detached from the school life and showing cynical attitude with other students, and sense of incapable, inferiority, helplessness and incompetent (Erschens et al., 2019).

The MBI-SS contains 15 items with a 7-point Likert scale ranging from never (0) to everyday (6). The internal consistency of total burnout, Emotional Exhaustion, Cynicism, and Inefficacy subscales using Cronbach’s alpha were reported as 0.867, 0.893, 0.870, and 0.837, respectively (Lee, Choi & Chae, 2017).

#### Temperament and Character Inventory

The TCI is Cloninger’s biopsychological personality measure comprising two-interrelated domains of temperament and character. Temperament traits are predisposed automatic responses to emotional stimuli and involuntary rational processes, while character traits are higher cognitive functions depicting one’s goals, values, and relationships with self, others, and the universe (Cloninger, 2008; Cloninger, Svrakic & Przybeck, 1993).

The temperament dimension includes four traits of Novelty Seeking (NS; characterized by exploratory excitability, impulsiveness, extravagance, and disorderliness), Harm Avoidance (HA; anticipatory worry, fear of uncertainty, shyness with strangers, and fatigability), Reward Dependence (RD; sentimentality, openness, attachment, and dependance), and Persistence (PS; eagerness, work-hardened, ambition, and perfectionism). The character dimension includes three traits of Self-Directedness (SD; characterized as purposeful, responsible, resourceful, and self-accepting), Cooperativeness (CO; empathic, helpful, forgiving, and tolerant), and Self-Transcendence (ST; contemplating, idealistic, spiritual, and transpersonal) (Cloninger, Svrakic & Przybeck, 1993; Lee et al., 2014; Min, Oh & Lee, 2007).

The Korean version of the TCI-Revised Short (TCI-RS) comprises 140 items with a 5-point Likert scale ranging from not at all (0) to very true (4). The internal consistencies of NS, HA, RD, PS, SD, CO, and ST were 0.83, 0.86, 0.81, 0.82, 0.87, 0.76, and 0.90, respectively (Min, Oh & Lee, 2007).

### Statistical analysis

The age difference between male and female students was analyzed using *t*-test, while the differences in education level and school year were analyzed using χ^2^ test. The gender differences in the TCI subscales and the MBI-SS subscales at three time points were investigated using *t*-test. The correlation between the TCI and MBI-SS at baseline was analyzed using Pearson’s correlation.

The General Growth Mixture Model (GGMM) was used to identify latent groups within the observed longitudinal data and to describe group differences in longitudinal changes between and within latent groups (Muthen & Muthen, 2000). Latent burnout developmental trajectories were extracted using the GGMM based on the MBI-SS total scores repeatedly measured at baseline, mid-term, and end-term.

The goodness of model fit used to determine the optimal number of latent trajectories in the GGMM was as follows (Muthén & Muthén, 2005): (1) one model is better when the Akaike Information Criterion (AIC), Bayesian Information Criterion (BIC), and adjusted BIC have smaller value than other models, (2) one model is better when Bootstrapped Likelihood Ratio Test (BLRT), Lo-Mendell-Rubin Likelihood Difference Test (LMR), or Vuong-Lo-Mendell-Rubin Likelihood Difference Test (VLMR) have more significant probability value than other models (Nylund, Asparouhov & Muthen, 2007), and (3) an Entropy index describing the classification quality of one model is greater than 0.8.

The differences in sex, age, school year, TCI subscales, and MBI-SS subscales among extracted latent burnout groups were examined using χ^2^ test and Analysis of Variance (ANOVA), and Scheffe or Dunnett’s T3 was used for post-hoc analysis depending on the result of Levene’s test for homogeneity of variance. The differences between baseline and end-term measures of the MBI-SS total scores in latent burnout groups were examined using paired *t*-test.

The differences in the MBI-SS total scores at baseline, mid-term, and end-term among the three extracted latent burnout groups were examined using ANOVA. Moreover, significant differences in the TCI subscale profiles among the three latent burnout groups were examined using profile analysis, and a Greenhouse–Geisser correction was incorporated when the results of Mauchly’s sphericity test were significant (Lee, Choi & Chae, 2017).

The data were presented as means and standard deviations or frequency and percentage. The *p*-values of 0.05, 0.01, and 0.001 were used for significance. All analysis were performed using IBM SPSS Statistics 25.0 (IBM, Armonk, NY, USA) except GGMM, which was analyzed using MPlus 5.21 (Muthen & Muhen, Los Angeles, CA, USA).

## Results

### Demographic features of participants

One hundred seventy-two participants (86 males and 86 females) completed MBI-SS three times (baseline, mid-term, and end-term of the semester) along with the TCI at baseline (Table 1).

**Table 1:** Demographic characteristics of the participants in current study.

		Male (*n* = 86, 50%)	Female (*n* = 86, 50%)	Total (*n* = 172, 100%)	*t* or χ^2^
Age**		30.35 ± 5.31	28.13 ± 3.54	29.38 ± 4.81	*t* = 3.230, *p* = 0.001
Education	Bachelor	61 (70.9%)	58 (67.4%)	119	χ^2^ = 0.245, *p* = 0.620
	Master	25 (29.1%)	28 (32.6%)	53	
Year	1st	27 (52.9%)	24 (47.1%)	51	χ^2^ = 5.590, *p* = 0.133
	2nd	26 (61.9%)	16 (38.1%)	42	
	3rd	18 (37.5%)	30 (62.5%)	48	
	4th	14 (48.4%)	16 (51.6%)	31	
MBI-SS	Baseline (1st week)				
	Total	48.69 ± 12.72	51.14 ± 12.7	49.91 ± 12.73	*t* = −1.266, *p* = 0.207
	Exhaustion	18.16 ± 6.48	19.1 ± 6.97	18.63 ± 6.73	*t* = −0.918, *p* = 0.360
	Cynicism	10.69 ± 5.27	11.27 ± 5.23	10.98 ± 5.24	*t* = −0.726, *p* = 0.469
	Inefficiency	19.84 ± 5.75	20.77 ± 4.73	20.3 ± 5.27	*t* = −1.158, *p* = 0.249
	Mid-term (9th week)				
	Total	53.55 ± 13.05	56.62 ± 14.17	55.08 ± 13.67	*t* = −1.478, *p* = 0.141
	Exhaustion*	19.35 ± 7.01	21.84 ± 7.4	20.59 ± 7.3	*t* = −2.263, *p* = 0.025
	Cynicism	12.5 ± 5.2	12.95 ± 5.89	12.73 ± 5.54	*t* = −0.535, *p* = 0.593
	Inefficiency	21.7 ± 6.03	21.83 ± 4.66	21.76 ± 5.37	*t* = −0.156, *p* = 0.877
	End-term (17th week)				
	Total	56.49 ± 15.96	58.79 ± 15.89	57.64 ± 15.92	*t* = −0.948, *p* = 0.345
	Exhaustion	20.71 ± 7.72	22.71 ± 8.03	21.71 ± 7.92	*t* = −1.665, *p* = 0.098
	Cynicism	14.22 ± 6.06	14.56 ± 6.34	14.39 ± 6.18	*t* = −0.357, *p* = 0.722
	Inefficiency	21.56 ± 6.51	21.52 ± 5.34	21.54 ± 5.93	*t* = 0.038, *p* = 0.969
TCI (baseline)					
	NS	33.52 ± 9.67	33.67 ± 10.66	33.60 ± 10.15	*t* = −0.097, *p* = 0.923
	HA	37.05 ± 10.48	39.40 ± 12.54	38.22 ± 11.59	*t* = −1.332, *p* = 0.184
	RD	45.05 ± 8.59	47.78 ± 9.62	46.41 ± 9.20	*t* = −1.965, *p* = 0.051
	PS	47.21 ± 9.42	47.23 ± 9.40	47.22 ± 9.38	*t* = −0.016, *p* = 0.987
	SD	51.09 ± 10.68	51.34 ± 9.97	51.22 ± 10.30	*t* = −0.155, *p* = 0.877
	CO	57.69 ± 8.55	58.79 ± 9.53	58.24 ± 9.04	*t* = −0.800, *p* = 0.425
	ST*	28.22 ± 12.17	32.49 ± 11.86	30.35 ± 12.17	*t* = −2.329, *p* = 0.021

**Notes:**

* *p* < .05.

** *p* < .01.

MBI-SS, Maslach Burnout Inventory-Student Survey; TCI, Temperament and Character Inventory; NS, Novelty-Seeking; HA, Harm-Avoidance; RD, Reward-Dependence; PS, Persistence; SD, Self-Directedness; CO, Cooperativeness; ST, Self-Transcendence.

Male students (30.35 ± 5.31) had a significantly higher age than female (28.13 ± 3.54) students did (*t* = 3.230, *p* = 0.001). There were no significant gender differences in education level (χ^2^ = 0.245, *p* = 0.620) and school years (χ^2^ = 5.590, *p* = 0.133). However, there were significant (*F* = 7.109, *p* < 0.001) differences between school years in Baseline measure of MBI-SS, and MBI-SS total score for 3rd year (55.15 ± 13.19) was significantly higher than that of 1st year (45.0 ± 11.36) in Scheffe post-hoc analysis.

Females (32.49 ± 11.86) showed significantly (*t* = −2.329, *p* = 0.021) higher score in ST than males (28.22 ± 12.17). Additionally, there were no significant differences in the MBI-SS subscale except the Emotional Exhaustion subscale at mid-term, which showed significantly (*t* = −2.263, *p* = 0.025) higher score among female (21.84 ± 7.4) than male students (19.35 ± 7.01).

### Correlations between subscales of MBI-SS and TCI at baseline

The correlations between the subscales of MBI-SS and TCI were examined using Pearson’s correlation (Table 2). The HA temperament was positively correlated with the MBI-SS total score (*r* = 0.283, *p* < 0.01) and Emotional Exhaustion (*r* = 0.217, *p* < 0.01), Cynicism (*r* = 0.177, *p* < 0.05), and Inefficacy (*r* = 0.230, *p* < 0.01) subscales. However, the SD character showed negative correlations with the MBI-SS total score (*r* = −0.386, *p* < 0.01) and Emotional Exhaustion (*r* = −0.228, *p* < 0.01), Cynicism (*r* = −0.302, *p* < 0.01), and Inefficacy (*r* = −0.341, *p* < 0.01) subscales. Along with these, the PS temperament correlated negatively with the MBI-SS total score (*r* = −0.196, *p* < 0.01) and Inefficiency (*r* = −0.321, *p* < 0.01) subscale. The MBI-SS Inefficacy subscale correlated negatively with NS (*r* = −0.165, *p* < 0.05) and RD (*r* = −0.228, *p* < 0.05) in the temperament domains.

**Table 2:** Correlation coefficient between subscales of MBI-SS and TCI subscales at baseline.

	TCI						
	NS	HA	RD	PS	SD	CO	ST
MBI-SS							
Total	−0.056	0.283***	−0.102	−0.196**	−0.386***	−0.112	−0.045
Exhaustion	−0.004	0.217**	0.015	−0.026	−0.228**	−0.024	0.027
Cynicism	0.034	0.177*	−0.039	−0.121	−0.302***	−0.112	−0.046
Inefficiency	−0.165*	0.230**	−0.228**	−0.321***	−0.341***	−0.127	−0.098

**Notes:**

* *p* < 0.05.

** *p* < 0.01.

*** *p* < 0.001.

TCI, Temperament and Character Inventory; NS, Novelty-Seeking; HA, Harm-Avoidance; RD, Reward-Dependence; PS, Pers stence; SD, Self-Directedness; CO, Cooperativeness; ST, Self-Transcendence; MBI-SS, Maslach Burnout Inventory-Student Survey.

Bold represents larger than 0.3.

### Extraction of latent burnout trajectory groups using the GGMM

**Figure 1:**
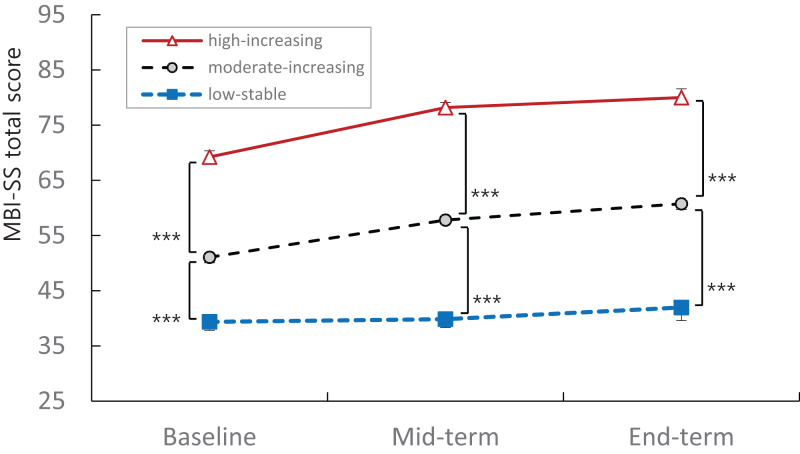
MBI-SS total score of three latent trajectories groups at baseline (1st week), mid-term (9th week) and end-term (17th week). The Maslach Burnout Inventory-Student Survey (MBI-SS) total score profiles of the three latent trajectories were not flat (Greenhouse–Geisser correction, df = 1.776, *F* = 26.605, *p* < 0.001) and the interaction of the three groups was significantly different as for the parallelism (Greenhouse–Geisser correction, df = 3.552, *F* = 4.318, *p* < 0.01). There was significant increase of MBI-SS total score between baseline and end-term in moderate (*n* = 99, *t* = 6.529, *p* < 0.001) and high (*n* = 22, *t* = 4.208, *p* < 0.001) burnout groups, however not in low burnout group (*n* = 51, *t* = 1.503, *p* = 0.139). The MBI-SS total scores of three latent groups at end-term were significantly different compared to those at baseline in moderate-increasing (*t* = 6.529, *p* < 0.001) and high-increasing (*t* = 4.208, *p* < 0.001) burnout groups, but not in low-stable burnout group (*t* = 1.503, *p* = 0.139). The high-increasing (12.8%) burnout trajectory group was shown using red triangle with solid line, the low-stable (29.6%) group using blue box with dotted line, and moderate-increasing (57.6%) group using gray circle with dotted line. Data shown as mean and standard error. ***p < 0.001.

Table 3 displays the GGMM model fit indices of the extracted latent burnout trajectories based on the MBI-SS total scores of participants at three time points. Three latent burnout trajectories were identified as shown in Fig. 1.

**Table 3:** General Growth Mixture Model fit indices for latent trajectory model based on BMI-SS total score of participants.

Model	AIC	BIC	adj. BIC	BLRT *p*	VLMR *p*	LMR *p*	Entropy
1-trajectory	4,200.074	4,218.959	4,199.96	N/A	N/A	N/A	N/A
2-trajectory	4,072.093	4,103.568	4,071.903	0	0.099	0.1072	0.708
**3-trajectory**	**3,998.128**	**4,042.193**	**3,997.862**	**0**	**0.002**	**0.0025**	**0.823**
4-trajectory	3,986.524	4,043.179	3,986.182	0.02	0.1899	0.2034	0.763
5-trajectory	3,984.038	4,053.282	3,983.62	0.1714	0.3867	0.3992	0.766

**Notes:**

GGMM, general growth mixture model; AIC, Akaike Information Criterion; adj., adjusted; BIC, Bayesian Information Criterion; BLRT, Bootstrapped Likelihood Ratio Test; LMR, Lo–Mendell–Rubin Likelihood Ratio Test; N/A, not applicable; VLMR, Vuong-Lo-Mendell-Rubin Likelihood Ratio Test.

Bold represents a selected trajectory model.

The three-trajectory model was selected as the proper model instead of the other trajectory models. The values of AIC (3998.128), BIC (4042.193), and adj.BIC (3997.862) of the three-trajectory model were smaller than those of one- and two-trajectory models, and the *p* values of BLRT (0), VLMR (0.002), and LMR (0.0025) of the three-trajectory model were more significant compared with those of the other trajectory models. The Entropy (0.823) of three-trajectory model was larger than 0.8, while that of the other models was smaller than 0.8.

### Academic burnout and personality characteristics of three trajectory groups

The Low-Stable (LS; *n* = 51, 29.7%), Moderate-Increasing (MI; *n* = 99, 57.6%), and High-Increasing (HI; *n* = 22, 12.8%) burnout trajectory groups were extracted and their demographic and psychological features were demonstrated in Table 4. There were significant differences in school year (Fisher’s Exact test = 17.073, *p* = 0.007) among three burnout trajectory groups, but not in sex (χ^2^ = 2.317, *p* = 0.344) and age (*F* = 0.178, *p* = 0.837).

**Table 4:** MBI-SS and TCI subscales of three latent burnout trajectory groups based on MBI-SS total scores at baseline, mid-term and end-term.

Latent burnout trajectories		Low-Stable (*n* = 51, 29.6%)	Moderate-Increasing (*n* = 99, 57.6%)	High-Increasing (*n* = 22, 12.8%)	Total (*n* = 172)	Post-hoc
	Male	28 (54.9%)	50 (50.5%)	8 (36.4%)	86 (50%)	χ^2^ = 2.317, *p* = 0.344
	Female	23 (45.1%)	49 (49.5%)	14 (63.6%)	86 (50%)	
Age		28.96 ± 0.48	29.29 ± 0.53	29.64 ± 0.88	29.24 ± 0.35	*F* = 0.178, *p* = 0.837
Year	1st	14 (27.5)	34 (66.7)	3 (5.9)	51	Exact test = 17.073, *p* = 0.007
	2nd	13 (31.0)	25 (59.5)	4 (9.5)	42	
	3rd	10 (20.8)	24 (50.0)	14 (29.2)	48	
	4th	14 (45.2)	16 (51.6)	1 (3.2)	31	
MBI-SS	Baseline					
	Total***	39.35 ± 7.94	51.06 ± 9.82	69.23 ± 7.15	49.91 ± 12.73	*F* = 86.621, LS<MI<HI
	Exhaustion***	14.33 ± 5.81	18.98 ± 5.48	27.05 ± 5.31	18.63 ± 6.73	*F* = 40.61, LS<MI<HI
	Cynicism***	7.16 ± 2.59	11.39 ± 4.67	17.95 ± 4.33	10.98 ± 5.24	*F* = 54.043, LS<MI<HI
	Inefficiency***	17.86 ± 4.61	20.69 ± 4.98	24.23 ± 5.36	20.3 ± 5.27	*F* = 13.552, LS<MI<HI
	Mid-term					
	Total***	39.84 ± 6.68	57.8 ± 6.67	78.18 ± 7.03	55.08 ± 13.67	*F* = 269.188, LS<MI<HI
	Exhaustion***	13.67 ± 4.49	22.13 ± 5.66	29.73 ± 4.58	20.59 ± 7.3	*F* = 83.238, LS<MI<HI
	Cynicism***	7.76 ± 2.85	13.42 ± 3.85	21.09 ± 5.14	12.73 ± 5.54	*F* = 99.55, LS<MI<HI
	Inefficiency***	18.41 ± 5.76	22.24 ± 3.88	27.36 ± 4.97	21.76 ± 5.37	*F* = 29.766, LS<MI<HI
	End-term					
	Total***	41.96 ± 11.26	60.75 ± 10.08	80 ± 11.02	57.64 ± 15.92	*F* = 109.781, LS<MI<HI
	Exhaustion***	14.76 ± 5.8	23.24 ± 6.34	30.91 ± 5.09	21.71 ± 7.92	*F* = 62.482, LS<MI<HI
	Cynicism***	9.47 ± 4.76	15.33 ± 4.89	21.55 ± 5.39	14.39 ± 6.18	*F* = 50.565, LS<MI<HI
	Inefficiency***	17.73 ± 5.22	22.17 ± 4.88	27.55 ± 6	21.54 ± 5.93	*F* = 29.923, LS<MI<HI
TCI						
	NS	33.71 ± 10.58	33.79 ± 10.03	32.50 ± 10.10	33.60 ± 10.15	*F* = 0.147
	HA***	32.69 ± 9.56	39.54 ± 10.66	45.14 ± 14.59	38.22 ± 11.59	*F* = 11.671, LS<MI, HI
	RD	47.69 ± 9.05	46.46 ± 8.35	43.23 ± 12.40	46.41 ± 9.20	*F* = 1.828
	PS*	49.73 ± 8.14	46.86 ± 8.66	43.05 ± 13.25	47.22 ± 9.38	*F* = 4.224
	SD***	56.86 ± 7.59	49.92 ± 9.45	43.95 ± 13.01	51.22 ± 10.30	*F* = 16.427, LS>MI, HI
	CO**	62.20 ± 8.88	56.22 ± 7.94	58.14 ± 11.23	58.24 ± 9.04	*F* = 7.948, LS>MI
	ST	30.94 ± 11.54	29.55 ± 12.41	32.64 ± 12.68	30.35 ± 12.17	*F* = 0.662

**Notes:**

* *p* < 0.05.

** *p* < 0.01.

*** *p* < 0.001.

TCI, Temperament and Character Inventory; NS, Novelty-Seeking; HA, Harm-Avoidance; RD, Reward-Dependence; PS, Persistence; SD, Self-Directedness; CO, Cooperativeness; ST, Self-Transcendence; MBI-SS, Maslach Burnout Inventory-Student Survey; LS, Low-Stable group; MI, Moderate-Increasing group; HI, High-Increasing group.

There were significant differences among the LS, MI, and HI burnout trajectory groups in the MBI-SS total score and three subscale scores at baseline, mid-term, and end-term (Table 4). Along with these, the MBI-SS total scores of the three latent groups at end-term were significantly higher compared with those at baseline in the MI (*t* = 6.529, *p* < 0.001) and HI (*t* = 4.208, *p* < 0.001) burnout groups but not in the LS burnout group (*t* = 1.503, *p* = 0.139).

There were significant differences in the developmental profiles of the MBI-SS total score among the three latent burnout groups (Fig. 1). The developmental profiles of the MBI-SS total score of the three latent trajectory groups were not flat (Greenhouse–Geisser correction, df = 1.776, *F* = 26.605, *p* < 0.001) and the interaction of the three groups was significantly different as for the parallelism (Greenhouse–Geisser correction, df = 3.552, *F* = 4.318, *p* = 0.003).

There were significant differences among the LS, MI, and HI burnout trajectory groups in the TCI subscales (Table 4). The HA score of the LS burnout group (32.69 ± 9.56) was significantly higher than those of the MI (39.54 ± 10.66) and HI (45.14 ± 14.59) burnout groups. The SD score of the LS burnout group (56.86 ± 7.59) was significantly higher than those of the MI (49.92 ± 9.45) and HI (43.95 ± 13.01) burnout groups. The LS (62.20 ± 8.88) burnout group also scored significantly higher in CO compared with the MI (56.22 ± 7.94) burnout group.

**Figure 2:**
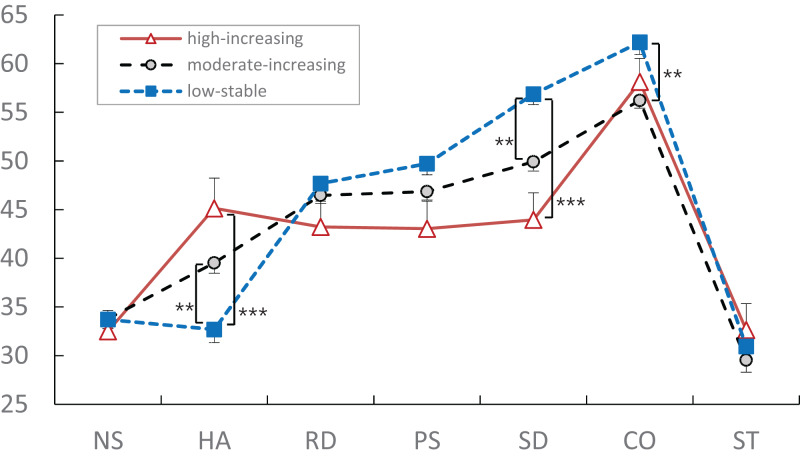
TCI subscale profiles of three latent academic burnout groups. The TCI subscale profiles of the three burnout latent trajectories were not flat (Greenhouse–Geisser correction, df = 4.469, *F* = 121.862, *p* < 0.001) and the interaction of the three trajectories was significantly different as for the parallelism (Greenhouse–Geisser correction, df = 8.939, *F* = 6.565, *p* < 0.001). The high-increasing (12.8%) burnout trajectory group was shown using red triangle with solid line, the low-stable (29.6%) group using blue box with dotted line, and moderate-increasing (57.6%) group using gray circle with dotted line. TCI, Temperament and Character Inventory; NS, Novelty-Seeking; HA, Harm-Avoidance; RD, Reward-Dependence; PS, Persistence; SD, Self-Directedness; CO, Cooperativeness; ST, Self-Transcendence. Data shown as mean and standard error. **p < 0.01; ***p < 0.001.

There was a significant difference in the TCI subscale profiles among the LS, MI, and HI burnout trajectory groups (Fig. 2). The TCI subscale profiles of the three burnout latent trajectory groups were not flat (Greenhouse–Geisser correction, df = 4.469, *F* = 121.862, *p* < 0.001) and the interaction of the three trajectories was significantly different as for the parallelism (Greenhouse–Geisser correction, df = 8.939, *F* = 6.565, *p* < 0.001).

## Discussion

The developmental trajectories of academic burnout among Korean medical students and personality profiles explaining the psychological characteristics of the longitudinal trajectories were analyzed using the GGMM and profile analysis. Three latent trajectory groups were extracted, and their personality profiles with HA (i.e., anticipatory worry, anxiety and fatigability) temperament and SD (i.e., maturity and self-esteem) and CO (i.e., empathy and tolerance) character dimensions were found pivotal in the current longitudinal study. These results were also consistent with the previous cross-sectional studies using single traits and personality profiles (Eley et al., 2016; Lee, Choi & Chae, 2017).

First of all, the current study extracted three latent burnout groups sharing a similar non-linear developmental trajectory (Hultell, Melin & Gustavsson, 2013; Martinent, Louvet & Decret, 2016; Salmela-Aro & Upadyaya, 2014): high-increasing (HI), moderate-increasing (MI), and low-stable (LS) groups. A person belonging to the vulnerable HI burnout group had a personality profile with a high score in HA (i.e., cautious, anxious and fatigable) and low scores in SD (i.e., immature and weak self-esteem) and CO (i.e., intolerant, opportunistic and revengeful) dimensions, while a person belonging to the resilient LS burnout group had a personality profile with a low score in HA (i.e., confident, optimistic and energetic) and high scores in SD (i.e., mature, purposeful and resourceful) and CO (i.e., empathic, helpful and principled) dimensions (Lee, Choi & Chae, 2017; Melchers et al., 2015; Yazici et al., 2014).

These results were consistent with the previous studies stating that those with a high burnout score appeared vulnerable to psychopathology along with a high score in HA and a low score in SD, while a person with a low burnout score appeared resilient along with a low score in HA and a high score in SD (Eley et al., 2016; Lee, Choi & Chae, 2017; Lee et al., 2014; Stoyanov & Cloninger, 2012). The combination of HA and SD is often emphasized for the psychopathological problems of patients (Cloninger, Bayon & Svrakic, 1998; Melchers et al., 2015; Miyoshi et al., 2016), stress recognition of healthy persons (Lee et al., 2014), and well-being and longevity (Cloninger & Zohar, 2011). That is, a multi-trait profile with the TCI dimensions of HA and SD within an individual and not a collection of inter-individual differences might be a realistic psychological configuration of a person, and could substantially influence the development of one’s mental health (Cloninger & Zwir, 2018).

Besides the clinical importance of HA and SD in academic stress of medical students, the current study displayed the importance of CO in explaining the developmental trajectory of academic stress: the LS latent trajectory group has significantly higher score in CO along with higher score in SD and lower score in HA than the MI latent trajectory group which was consistent with the resilient group in the previous study (Eley et al., 2016).

The CO subscale represents the understanding of the self as integral for humanity and society with subscales of empathic, helpful, forgiving, and tolerant (Eley et al., 2016). And it might be pivotal for suppressing stress and its negative effects in the medical field, considering that the social relationship and support have a positive effect in ameliorating long-term academic stress of medical students.

The CO trait might influence professional attitude, patient-doctor relationship, and the quality of the medical practice of clinicians after graduation (Lee et al., 2014). The clinician’s emotional distance (an aspect of a low CO score) towards the patient is deliberately introduced as a professional attitude for the efficacy of medical treatment and therefore the depersonalization and cynicism tend to be its undesired and negative side effects. Nurturing the CO trait might solve these problems, and improve the biopsychological well-being and quality of life in medical education and professional career.

The character development (e.g., SD and CO traits) seems important in medical education where the character can control a genetically predisposed temperament (e.g., HA trait). The SD character dimension representing the purposeful, resourceful, and self-actualizing characteristics of medical students has often been emphasized in medical education. Additionally, fostering of CO character may replenish the empathic and warm stance towards patients, a cooperative and principled attitude towards fellow clinicians, and a helpful and forgiving perspective towards society (Eley et al., 2016). Studies on medical education frequently advised the inclusion of courses for securing mental health of medical students such as mind-body skill programs, wellness programs, mentoring programs, learning relaxation techniques and social activities, and learning and practicing adaptive coping styles emphasizing problem-solving and asking for help (Frajerman et al., 2019). Consistent with the previous suggestions, for example, the well-being coach program of the Anthropedia Foundation developed for cultivating the TCI character dimensions of SD and CO might be useful for improving the well-being of medical professionals (Cloninger et al., 2019b; Lee et al., 2014).

This study has some limitations concerning the generalization of the findings. First, since the data for the study were collected from medical students, the characteristics and mechanism of academic stress might be different from ordinary college students or high school students. It may be because the academic stress and burnout symptoms of medical students are mainly associated with worries and anxiety concerning possible failure along with the tremendous volume of learning and frequency of examinations. Compared to medical students, the psychological burden of college and high school students might be less considering relatively small amount of learning and examination. Future studies should include college students or high school students to generalize the results.

Second, the influence of occupation, duration, and type of medical education might mediate the influence of personality on academic stress. Future studies should include participants from nursing schools, with a high ratio of female students, and clinicians from internship and residency training. Moreover, the long-term effect of 4 or 6 years of medical education and the mediating effect of winter break and summer vacation might also require investigation concerning the resilience of medical students.

Third, the number of participants per year was limited in the current study and the effects of school year should be examined with bigger sample size. The students of 3rd year have higher baseline MBI-SS total score than the 1st year students, and have higher distribution of HI latent burnout trajectory group than others. These results might come from the amount of assigned learning since the first semester of 3rd year has overwhelming volume of theories and knowledge needed for the clinical observation and bedside teaching planned for the next semester. In the current study, the clinical exposure has no significant influence on the magnitude of burnout symptoms.

Last but not the least, the high variance of the TCI subscale measures in the HI trajectory group made the use of nonparametric post-hoc analysis and subsequently found indistinctive differences in TCI subscales between the trajectory groups. Hence, future studies with a large sample size might decrease the variance and present the explicit differences in the TCI between latent groups.

## Conclusion

The current study analyzed the effect of personality profiles on the developmental trajectories of latent academic burnout in Korean medical students using the GGMM. The high-increasing burnout trajectory group displayed a higher score in HA and lower scores in SD and CO, while the low-stable burnout trajectory group revealed a lower score in HA and higher scores in SD and CO. These results indicate the influence of personality traits on the mental health of medical students: burnout symptoms of medical students might not be temporary symptoms from academic stress but longstanding symptoms stem from their personality profiles. The psychological health of students should be looked after actively during the medical school years, which may be beneficial to the doctor-patient relationship and the quality of clinical practice in the future.

## Supplemental Information

10.7717/peerj.10362/supp-1 Supplemental Information 1 Raw data.

## Additional Information and Declarations

### Data Availability

The following information was supplied regarding data availability: The raw measurements are available as a Supplemental File.
